# Study protocol: improving the transition of care from a non-network hospital back to the patient’s medical home

**DOI:** 10.1186/s12913-017-2048-z

**Published:** 2017-02-10

**Authors:** Roman A. Ayele, Emily Lawrence, Marina McCreight, Kelty Fehling, Jamie Peterson, Russell E. Glasgow, Borsika A. Rabin, Robert Burke, Catherine Battaglia

**Affiliations:** 1 Department of Veterans Affairs, Eastern Colorado Health Care System, 1055 Clermont Street, Research (A151), Denver, CO 80220 USA; 2 University of Colorado, Anschutz Medical Campus, Aurora, CO USA; 3 Department of Family Medicine and Public Health, School of Medicine, University of California San Diego, La Jolla, California USA; 4 Department of Family Medicine, School of Medicine, University of Colorado, Aurora, Colorado USA

## Abstract

**Background:**

The process of transitioning Veterans to primary care following a non-Veterans Affairs (VA) hospitalization can be challenging. Poor transitions result in medical complications and increased hospital readmissions. The goal of this transition of care quality improvement (QI) project is to identify gaps in the current transition process and implement an intervention that bridges the gap and improves the current transition of care process within the Eastern Colorado Health Care System (ECHCS).

**Methods:**

We will employ qualitative methods to understand the current transition of care process back to VA primary care for Veterans who received care in a non-VA hospital in ECHCS. We will conduct in-depth semi-structured interviews with Veterans hospitalized in 2015 in non-VA hospitals as well as both VA and non-VA providers, staff, and administrators involved in the current care transition process. Participants will be recruited using convenience and snowball sampling. Qualitative data analysis will be guided by conventional content analysis and Lean Six Sigma process improvement tools. We will use VA claim data to identify the top ten non-VA hospitals serving rural and urban Veterans by volume and Veterans that received inpatient services at non-VA hospitals.

Informed by both qualitative and quantitative data, we will then develop a transitions care coordinator led intervention to improve the transitions process. We will test the transition of care coordinator intervention using repeated improvement cycles incorporating salient factors in value stream mapping that are important for an efficient and effective transition process. Furthermore, we will complete a value stream map of the transition process at two other VA Medical Centers and test whether an implementation strategy of audit and feedback (the value stream map of the current transition process with the *Transition of Care Dashboard*) versus audit and feedback with Transition Nurse facilitation of the process using the Resource Guide and Transition of Care Dashboard improves the transition process, continuity of care, patient satisfaction and clinical outcomes.

**Discussion:**

Our current transition of care process has shortcomings. An intervention utilizing a transition care coordinator has the potential to improve this process. Transitioning Veterans to primary care following a non-VA hospitalization is a crucial step for improving care coordination for Veterans

## Background

Care coordination can be highly problematic for health systems to re-establish care with the patient’s medical home when patients transition across multiple systems. Deficient care coordination processes often results in adverse clinical events due to fragmented care, lack of communication between providers and patient confusion regarding post-discharge medications and timing of follow-up care [1–5]. In 2011, poorly managed transitions were estimated to cost between $25 to $45 billion due to avoidable complications and unnecessary hospital readmissions [6]. Transitions are also vulnerable exchange points for patients and caregivers, especially for older patients coping with multiple co-morbidities and complex regimens. Patients have identified coordination of care as one of the factors that influences their perception of quality [7]. Care coordination tools such as clinical pathways, information systems, case management, as well as high-quality communication and strong relationships among health care providers need to be available to provide efficient clinical outcomes, and the exchange of critical information among providers [7].

There are several reasons for poor care coordination in the transition from hospital back to primary care. First, different electronic medical record systems make it difficult to transmit medical information between hospitals and physician practices. Consequently, the primary care providers lack information about their patients’ hospitalizations [8, 9]. A study in 2007 found that only 12–34% of primary care providers had received a hospital discharge summary about their patients’ hospitalization and had it on hand during their patient’s follow-up appointment. Even when discharge summaries are received, they often lack key information, such as test results, treatment course, discharge medications, and follow-up plans further contributing to ineffective care coordination [8].

Providing safe and comprehensive care coordination to Veterans who access care across multiple systems is challenging for the Department of Veterans Affairs (VA). Problems found in other health systems are not uncommon when Veterans are hospitalized in a non-VA hospital and transition back to VA primary care for follow-up. Care coordination tools that ensure a smooth transition are not consistently available. Given that VA hospitals tend to be located in urban areas, Veterans who live further from a VA facility are less likely to rely on the VA for inpatient care [9, 10]. This increases the Veteran’s chance of receiving care at a non-VA hospital. Delivering comprehensive quality health care for these Veterans is often dependent upon the coordination and integration of VA health care services with non-VA care systems.

### Objective

The goal of this transition of care quality improvement (QI) project is to identify gaps in the current transition process and implement an intervention that bridges the gap and improves the current transition of care process within the Eastern Colorado Health Care System (ECHCS). We will use value stream mapping (a Lean Six Sigma tool) to explore the current steps a patient must take to transition from non-VA hospital to VA primary care [11]. Moreover, we will identify inefficiencies in the current process, implement an intervention to provide effective, coordinated care in a team-based environment, and test the effectiveness and sustainability of the intervention.

### Conceptual framework and theoretical foundation

A comprehensive conceptual model called a Practical, Robust Implementation and Sustainability Model (PRISM) will be used for integrating research findings into practice. Rooted in concepts from QI, the Chronic Care Model, the Diffusion of Innovations theory, and measures of population-based effectiveness of translation, PRISM evaluates how a healthcare program or intervention interacts with the recipients to influence program reach, adoption, implementation, maintenance, and effectiveness [12]. Main domains of PRISM include Organizational characteristics, Patient/provider characteristics, Intervention from the perspective of the organization and patient, external environment, and sustainability infrastructure.

**Fig. 1:**
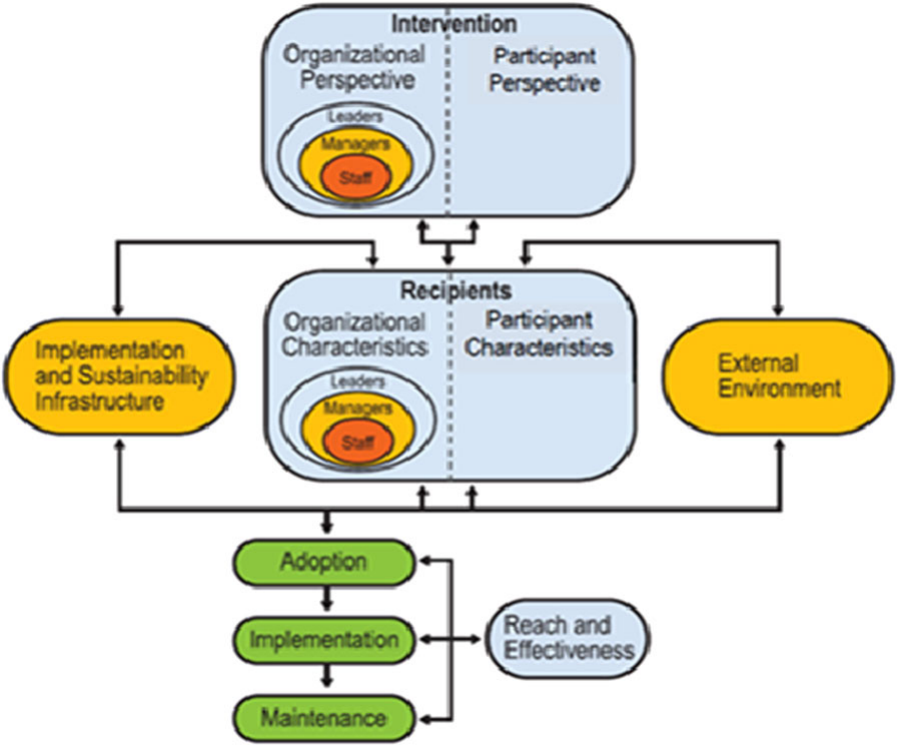
The PRISM Framework used to guide planning, implementation, and evaluation of the QI project and to frame the implementation core

PRISM (Fig. 1) PRISM highlights four components that influence implementation success: 1) organizational and participants characteristics; 2) intervention characteristics from the organizational (medical facility) and participants’ perspectives (i.e., patients and providers); 3) implementation and sustainability infrastructure (training and support); and 4) external environment. PRISM also identifies a set of important outcomes from the RE-AIM model (i.e., Reach and Effectiveness, Adoption, Implementation, and Maintenance) for evaluation. Important elements to improve program implementation based on PRISM include creating an environment for encouraging spread, sharing best practices, observing results and adjusting processes accordingly, facilitating use of the intervention, and ensuring adaptability of protocols. These elements will be used in a formative manner and incorporated into our planning and implementation process. Key outcomes and analyses related to the program and implementation strategy will be evaluated using the Reach, Effectiveness, Adoption, Implementation and Maintenance (RE-AIM) measures [13].

We will also use Haggerty’s Continuity of Care (CoC) Framework [14] and the principles established in the Transitions of Care Consensus Policy Statement [15], to define an ideal transition of care process. The ideal transitions of care process will address the domains referenced in Table 1 that are associated with improved care transitions [16]. These ideal transitions of care domains will help us guide our intervention to improve the current process and to make it ideal.

**Table 1:** Key components of an ideal transition in care

Domains	Description
Discharge Planning	Involves the important principle of planning ahead for hospital discharge while the patient is still being treated in the hospital.
Complete communication of information	Refers to the content that should be included in discharge summaries and other means of information transfer from hospital to post-discharge care.
Availability, timeliness, clarity and organization of information	Important because post-discharge providers must be able to access and quickly understand the information they have been provided before assuming care of the patient.
Medication Safety	This is of central importance because medications are responsible for most post-discharge adverse events.
Educating patients to promote self-management	Involves teaching patients and their caregivers about the main hospital diagnoses and instructions for self-care, including medication changes, appointments, and whom to contact if issues arise.
Coordinating care among team members	This is needed to synchronize efforts across settings and providers.
Monitoring and managing symptoms after discharge	Monitoring for new or worsening symptoms; medication side effects, discrepancies, or nonadherence; and other self-management challenges will allow problems to be detected and addressed early, before they result in unplanned healthcare utilization.
Outpatient Follow-up	Optimal follow-up with appropriate post-discharge providers is crucial for providing ideal transitions. These appointments need to be prompt (e.g. within 7 days if not sooner for high-risk patients) and with providers who have a longitudinal relationship to the patient, as prior work has shown increased readmissions when the provider is unfamiliar with the patient

## Methods and design

### Project description and study design

This project has three phases. During the first phase (Aim 1) we will: 1) interview key VA providers and staff informants and Veterans hospitalized in a non-VA hospital in 2015 and 2) interview non-VA providers and staff informants from high volume urban and rural hospitals used by ECHCS Veterans. The goals of Aim 1 are to: 1) create a value stream map with current processes and develop an ideal transition of care process, 2) identify gaps in transition of care process between current and ideal processes of care, and 3) create a *Transition of Care Resource Guide* for VA and non-VA providers as well as patients that would allow for monitoring and feedback of the process subsequent phases to ensure optimal continuity of care. Phase 1 will be completed during the first year of the project.

During the second phase (Aim 2) we will: 1) develop and pilot test an intervention utilizing a Transition Nurse role and system changes to facilitate continuity of care for Veterans returning to VA primary care at ECHCS after non-VA hospitalization. The goal of Aim 2 is to test the intervention using repeated improvement cycles incorporating factors that are identified as value-added based on data collected of identified process measures. These data will be monitored and displayed on the *Transition of Care Dashboard*. We will have both process and outcomes measures such as number of non-VA hospital discharge summaries received within 14 days, number of post discharge follow up visits with VA primary care provider within 14 days of discharge, ER utilization rates 30 days after hospital discharge and re-hospitalization rates 30 days after hospital discharge. Phase 2 will be completed during the second year of the project.

**Fig. 2:**
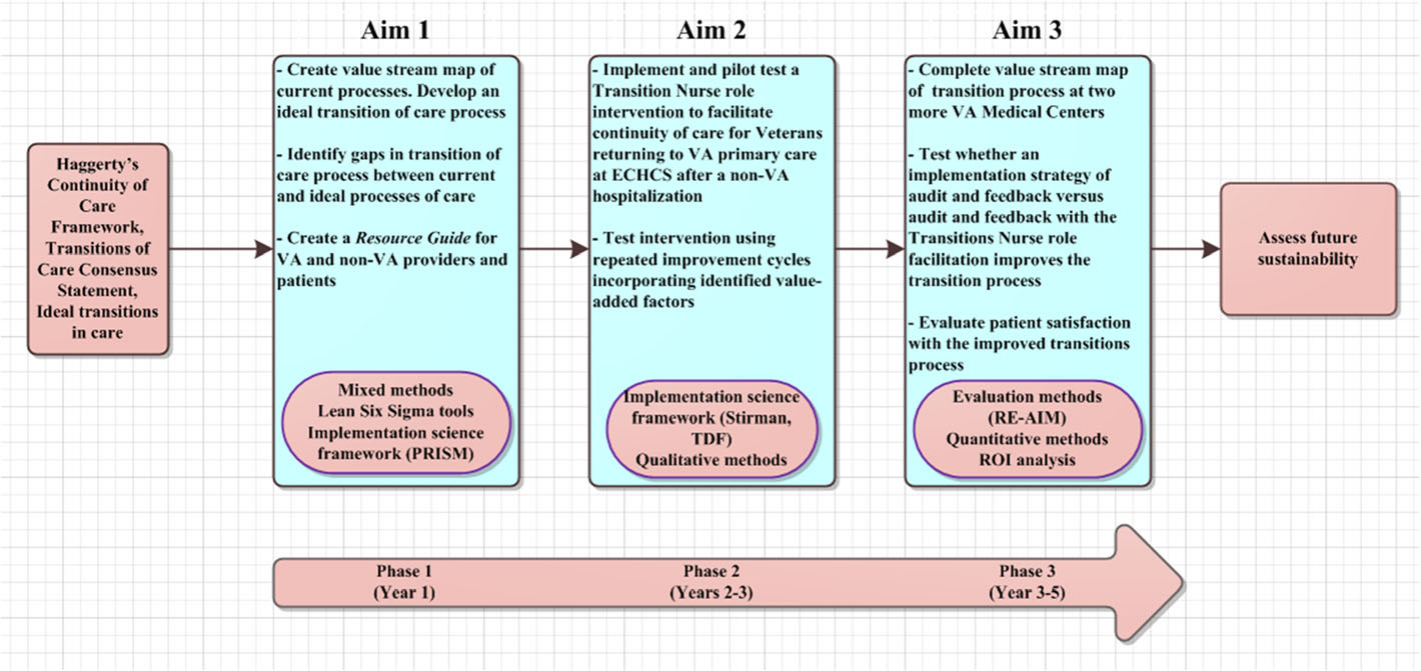
Transitions of Care project description

During the third and final phase (Aim 3) we will: 1) complete a value stream map of the transition process at two other VA Medical Centers in Veterans Integrated Service Networks (VISNs) 19 in consultation with the VISN 19 Chief Medical Officer and 2) test whether an implementation strategy of audit and feedback (the value stream map of the current transition process with the *Transition of Care Dashboard*) versus audit and feedback [17, 18] with Transition Nurse facilitation of the process using the Resource Guide and Transition of Care Dashboard improves the transition process, continuity of care, patient satisfaction and clinical outcomes. The Transition Nurse will provide facilitation using the Transition of Care Resource Guide and a Transition of Care Dashboard. This project was reviewed by the Colorado Multiple Institutional Review Board and deemed to be a QI project (See Fig. 2).

#### Partnership approach

##### Evaluation team

Our multidisciplinary evaluation team consists of experts in the field of qualitative and quantitative research, Lean Six-Sigma, statistics and data management, implementation science, public health, nursing and medicine who bring together experience, knowledge, methodological rigor and skills for the success of this QI project.

##### Operational partners

This project will be carried out in partnership with Office of Community Engagement, ECHCS stakeholders, VISN 19, Rural Health Resource Center-Western Region, the VA Office of Community Care as well as the Quality Enhancement Research Initiative (QUERI) Program which is providing protected time for all the personnel to participate in all aspects of this QI project. This partnership enables us to implement the proposed study design, evaluate the intervention’s impact, and disseminate findings to other sites. The proposed Transition Nurse role is intended to augment current programs by ensuring care coordination for hospitalized Veterans returning to VA primary care.

##### Relationship between evaluation team and operational partners

The multidisciplinary team has a longstanding presence in conducting Veteran-centered research with expertise in program evaluation, quality improvement initiatives and implementation research. The team is composed of individuals with experience providing scientific and methodological expertise, as well as QI efforts in their respective focus areas (medicine, public health, nursing, research). To ensure continued value and relevance of the project and engagement of key stakeholders with the project, a Strategic Advisory Group is in place to provide feedback, guidance and assistance for engagement with project sites throughout the project. The Strategic Advisory Group is composed of VA and non-VA leaders and key stakeholders.

#### Key informant interviews

We will conduct in-depth, semi-structured key-informant interviews pre-intervention development with VA and non-VA providers and staff informants as well as Veterans to understand the current transition of care, facilitators and barriers of the processes and obtain suggestions for improvement. The semi-structured interview guides will be aligned with domains from PRISM such as *Program*/*intervention*-*organizational perspective*, *Program*/*Intervention*- *Patient perspective*, *Implementation and Sustainability Infrastructure* and *Characteristics of Organizational recipients*. Utilization of the PRISM model will assist in the assessment of the different organizational perspectives, characteristics of intervention recipients and translation of qualitative findings into a sustainable intervention. Participants will be recruited using convenience and snowball sampling from VA and non-VA hospitals frequented most by Veterans. Qualitative data analysis will be done using Atlas.ti software. Information obtained from the qualitative inquiry along with the results of Lean Six Sigma assessment will inform the development of the current transition process map and the value-stream map. Findings from the key informant interviews will help in designing an intervention to improve the existing transition process.

#### Qualitative data analysis

Utilize a conventional content analysis [19] technique for qualitative data analysis, interviews will be conducted by evaluation team members and transcribed verbatim. Two analytic team members will independently code the initial interview for each role interviewed and meet to review the documents to clarify meanings of codes and come to consensus when disagreements occur, thus defining the initial code book. Emergent codes will be added to the code book as they come up [20–22]. Consensus building will be achieved by discussing coding consistency with the qualitative evaluation team and resolution of differences in coding among the evaluation team members. All transcripts will then be coded using Atlas.ti. This process will be repeated as interviews take place for each new site. The coded data will then be examined and organized into categories, domains, or themes. We will continue this process until no new concepts are identified. Results will be reviewed by members of the evaluation team to assess thoroughness and comprehensiveness [20, 21].

#### Analyses of Lean Six Sigma

As part of the Lean Six Sigma approach, we will describe the components of the Define, Measure, and Analyze (DMAIC) assessment to analyze the current transition of care process. Initially, we will create a value stream map identifying current steps, documentation of time at each step, delays, and information flows required to deliver specific components of the transition process. This will help identify gaps in processes, target improvement priorities, and construct a roadmap to close gaps between ideal and current transition of care process map. Next, using the results of the qualitative data analysis, we will create a detailed process map of the steps Veterans take post discharge from a non-VA hospital to VA primary care. This will help visualize value added and non-value added activities in the current transition process.

Furthermore, we will utilize ideal process descriptions from key informant interviews and literature to map the ideal transition of care process. This will help visualize the gaps in transition of care process between the current and ideal process as well as prioritize targets for improvement. To conceptualize, develop and evaluate the intervention based on our understanding of the current transition process, we will identify activities, resources, and external factors that could influence the results. This will help us capture expected outcomes of the different intervention activities and ensure the intervention is aligned with the original intent of our study.

#### Development of transition of care resource guide

We will create a *Transition of Care Resource Guide* for VA and non-VA providers and Veterans to ensure implementation of the intervention. The Resource Guide will consist of two checklists: 1) provider checklist will identify physician name/contact information at non-VA hospital, discharge diagnosis/discharge plan, outstanding lab/imaging/pathology results as well as critical or high risk medication(s), and when the patient needs follow-up care. This checklist and medical records will be sent to VA primary care providers by non-VA hospitals; and 2) patient checklist that will provide information on follow-up care and key contact information at the VA. The resource guide items will allow for monitoring and feedback of the process during rollout in the next phase to ensure that pertinent information is received by VA primary care and that Veterans are receiving optimal continuity of care.

There are two major goals we want to accomplish in Aim 3. First we will implement and test the Transitions Nurse role in the field using repeated improvement cycles. Potential solutions will need to promote greater standardization to an otherwise fragmented delivery system. The different intervention elements of the Transitions Nurse role will be determined by what we learn about the current transition process from the qualitative interviews and Lean Six Sigma tools. We have successfully implemented a Transition Nurse role for Veterans who are referred from VA facilities/clinics to Denver VA for inpatient specialty care. Use of the Transition Nurse role has led to decrease in emergency department visits in the 30 days after hospital discharge with high provider and patient satisfaction [23].

Based on the review of the literature, some of the ways the Transition Nurse will facilitate implementation of the new processes include: 1) Collaborating with the non-VA discharge planners/case managers to complete needed documentation and/or medical record transfer. This provides VA primary care with essential details of the hospitalization and follow-up plan; 2) Obtaining follow-up appointment at the VA primary care within 14 days of discharge or sooner if clinically indicated; 3) Calling the Veteran within 72 hours after discharge to assess symptoms and concerns, perform medication reconciliation, verify planned follow-up appointment attendance, and assess discharge status (knowledge of self-care, medications, whom to contact, and health literacy) of the patient [24]. The Transition Nurse will use teach-back methodology to address gaps in discharge preparedness [25] and will remain available as a resource for the patient until the Veteran is reintegrated into VA primary care. If a Veteran does not have an assigned primary care provider, the Transition Nurse will facilitate getting a VA primary care assignment and follow-up.

The second goal of years 3–5 is to conduct a value stream map of the transition process at two other VA Medical Centers in VISN 19. We will test whether an implementation strategy of audit and feedback (of the value stream map of the transition process) versus audit and feedback with nurse facilitation improves the transition process, continuity of care, satisfaction and clinical outcomes. We will scale up the evidence based intervention to two other VA medical centers. We will create a value stream map of their process, adapt the *Transitions of Care Resource Guide* to the local context of the Medical Centers and include site specific information. Then, we will work with each Medical Center to implement a Transition Nurse role or identify someone currently at the Medical Center who can fulfill the role. For all aims, we will use the Stirman Framework [26] to systematically track adaptations and modifications to both data gathering methods and the formation and adaptation of the intervention and its implementation.

#### Evaluation of intervention

We will evaluate our intervention and implementation strategy using the RE-AIM measures. RE-AIM measures are a common evaluation approach to assess the impact of the implementation strategies and sustainability.

**Reach** is defined in terms of the proportion of Veterans hospitalized at non-VA facility transitioning back to VA primary care. We will assess the absolute number, proportion, and representativeness of VA primary care, non-VA providers and patients who receive the *Transition of Care Resource Guide* checklist during the transition.

**Effectiveness** of the intervention will be evaluated by the following metrics: 1) adherence to *Transition of Care Resource Guide* on a monthly basis. We will set-up a collaborative teleconference with a plan for scheduled on-line meetings to allow stakeholders to talk through issues and problem solve as a group; 2) satisfaction of the patients’ transition experience using the validated Care Transition Measure (CTM) [27, 28] and Likert-scale questions to assess satisfaction, which will reflect the overall quality of transition process (8–12 calls/month based on thematic saturation); 3) satisfaction of the providers (VA and non-VA) experience through key informant interviews; and 4) emergency department and re-hospitalizations in the 30 days after index hospital discharge from the non-VA hospital.

**Adoption** is the absolute number, proportion, and representativeness of settings and intervention agents (Medical Centers) who are willing to participate in the Transition Nurse program. Furthermore, adoption of the Transitions Nurse program internally at the Denver VA medical center and by participating patients will be evaluated.

**Implementation** is defined as the extent to which the intervention is implemented as intended as well as adaptations made to the intervention. We will focus on barriers and facilitators of the implementation and assess adaptations and modifications to the intervention using Stirman’s framework [26]. We will also conduct a standardized Return on Investment analysis to assess intervention cost-benefit and future sustainability. Through the Lean Six Sigma process, we will seek to understand how organizational context affects QI efforts, organizational context such as staffing, culture, teamwork, leadership, and communication [29, 30] have all influenced the uptake and sustainability of QI improvements. Because organizational context is important, we will collect data through interviews and field notes (during collaborative teleconferences) about job tasks, roles, and social dynamics. Additional details are in the implementation core section.

**Maintenance**: Maintenance will be assessed via long-term use of *Transitions of Care Resource Guide* and extent to which sites continue the Transition Nurse program to facilitate Veterans transition following hospital discharge. Furthermore, we will also evaluate the extent of patients continuing to participate in the Transitions Nurse program

## Discussion

### Potential challenges and limitations

This project aims to evaluate the current transition of care process and implement an intervention to promote smooth transition for Veterans hospitalized in a non-VA hospital and who return to VA for follow-up primary care. We will work with operational partners to identify deliverables viewed as having an important impact on improving the transition of care process for Veterans. These include: 1) Provide value stream map of processes that promotes continuity of care and create a *Transition of Care Resource Guide* with “ideal” transition processes and checklists; 2) An evaluation of the different evidence-based transitions of care approaches including the Transition Nurse will be added to the *Transition of Care Resource Guide* with templates for dashboards and interview guides, and 3) An Evaluation Report using the RE-AIM measures of the two implementation strategies to improve the transition process, continuity of care, and clinical outcomes following out of VA hospitalization will be completed.

A few limitations may arise during this project. First, different target audiences might have different reactions to using a Resource Guide which might not be updated regularly to accommodate the changing needs of different organizations (VA and non-VA). Another potential challenge lies with sustainability of a Transitions Nurse due to the cost associated with an additional FTE. The Return on Investment evaluation will provide valuable information about cost effectiveness of the intervention. Finally, developing an intervention to improve care transitions assumes that non-VA hospitals are able and willing to participate in improving the current process. This may limit the intervention adaption and outcomes of the intervention.

### Contribution to practice

This project will contribute to the improvement of transitions of care processes for Veterans. As a result of the 2014 CHOICE Act, which provided eligible Veterans an opportunity to receive non-VA care, the number of Veterans receiving care in a non-VA hospital is increasing. Understanding the barriers and facilitators to the current transition of care process for these Veterans when they return to VA primary care is essential to ensure proper follow-up care, medication reconciliation, and transfer of information from non-VA hospital settings. Effective implementation of this project will help Veterans in one of the most vulnerable times post discharge from non-VA hospitalizations. This project is addressing an area that has not been evaluated in previous projects at ECHCS with input from interdisciplinary teams, local and national stakeholders.

## Abbreviations

ECHCS: Eastern Colorado Health Care System
PRISM: Practical, Robust Implementation and Sustainability Model
TDF: Theoretical domains framework
VA: Veterans Affairs
VA HSR&D: Veterans Affairs Health Services Research and Development
